# Real-World Efficacy and Safety of Tafluprost in Primary Open-Angle Glaucoma Patients with Corneal Disorders: A Taiwan Experience

**DOI:** 10.1155/2022/4885485

**Published:** 2022-10-29

**Authors:** Ju-Kuo Lin, Fu-Tsung Wei, Yi-Chun Liu, Yung-Chang Yen

**Affiliations:** ^1^Chi Mei Medical Center, No. 901, Zhonghua Road, Yongkang, Tainan 71004, Taiwan; ^2^Chi Mei Medical Center, Liouying, No. 201, Taikang, Taikang Village, Liouying, Tainan 73657, Taiwan

## Abstract

**Purpose:**

To investigate the efficacy and safety of switching to 0.0015% tafluprost ophthalmic solution with reduced benzalkonium chloride (BAK) on primary open-angle glaucoma (POAG) patients with corneal disorders under 0.005% latanoprost treatment. *Material and Methods*. This was a single-arm, open-label, switching study on adult POAG patients treated with latanoprost 0.005% for more than 3 months, with corneal disorders but no dry eye therapy. All patients were switched to tafluprost 0.0015% and followed up for 3 months. The primary outcome was the change in fluorescein staining score (National Eye Institute/Industry [NEI] score) at the end of the study. Secondary outcomes included changes in intraocular pressure (IOP), tear break-up time (TBUT), hyperemia score, and other ocular and nonocular adverse events.

**Results:**

Of the 20 patients initially enrolled, 17 patients, all with POAG, completed the study. At the end of the study, the mean NEI score significantly decreased by 1.8 ± 2.2 (*p* < 0.01). No significant changes in IOP were observed (12.8 ± 4.6 mmHg at baseline vs. 12.3 ± 4.0 mmHg on visit 2; *p*=0.470). TBUT increased by 1.2 ± 1.7 seconds (*p* < 0.05). The proportions of patients with no sign of hyperemia on the bulbar and palpebral conjunctiva increased from 58.5% to 64.7% at baseline (before switching to tafluprost treatment) to 94.1% and 94.1%, respectively, after switching to tafluprost treatment. Dry eye sensation scores were significantly reduced (*p* < 0.05), while other ocular symptom scores did not change significantly.

**Conclusion:**

Switching to tafluprost 0.0015% significantly improved fluorescein staining score, TBUT, and conjunctival hyperemia while maintaining IOP control among POAG patients with corneal disorders.

## 1. Introduction

Glaucoma is a chronic and progressive ocular disorder characterized by optic neuropathy (enlarged optic disc cupping, loss of the neuroretinal rim, and thinning of the retinal nerve fiber layer) and deterioration of the visual field. The latest data in Taiwan (2010) showed that the crude incidence rates of primary open-angle glaucoma (POAG) and angle-closure glaucoma were 2.44 and 65.97 per 100,000 persons, respectively [1].

The treatment for glaucoma includes therapies that lower intraocular pressure (IOP), such as medication, laser, surgery, or a combination of approaches. Medications are considered the first-line treatment for glaucoma [2]. The reduction of IOP has been demonstrated to slow down or halt disease progression [3–5].

Prostaglandin analogues (PGAs) are commonly used IOP-lowering medications in Taiwan. Since their introduction, PGAs such as latanoprost and travoprost have grown to become some of the most prescribed IOP-lowering medications in Taiwan [6]. However, these drugs have been associated with ocular surface diseases (OSDs), probably due to the effects of prolonged exposure to benzalkonium chloride (BAK), a commonly used preservative for PGAs [7–9]. Taiwanese data showed that the risk of dry eye was increased in patients treated with PGAs (odds ratio 1.48; 95% CI 1.30, 1.69) [10].

Tafluprost is a newer PGA with a 12-fold higher affinity to the prostaglandin FP receptor compared with latanoprost. Commercially available tafluprost ophthalmic solution contains a lower concentration of BAK compared with latanoprost ophthalmic solution [11].

BAK is a common preservative in multi-dose formulations; however, OSDs, due to the chronic use of BAK-containing antiglaucoma medications, may ameliorate after switching to formulations with less BAK or without BAK. An investigation of epithelial permeability and autofluorescence of the cornea in patients with POAG or ocular hypertension (OH) demonstrated that removal of BAK from timolol resulted in an improvement of corneal epithelial barrier function and in a reduction of complaints [12]. On the other hand, it was reported in 75% of glaucoma patients that the superficial punctate keratopathy (SPK) scores improved by switching their glaucoma drugs (either latanoprost or travoprost) to BAK-reduced tafluprost [13]. Besides, switching therapy from BAK-preserved latanoprost to travoprost without BAK significantly reduced the frequency of SPK in POAG or OH patients [14].

In our practice, we tended to limit the use of concomitant ocular medications to no more than three medications. Prevention of OSDs in patients on topical glaucoma therapy can be achieved by reducing exposure to BAK. This study aimed to investigate the effect of 0.0015% tafluprost ophthalmic solution with reduced BAK on patients with POAG and corneal disorders under 0.005% latanoprost treatment.

## 2. Materials and Methods

### 2.1. Study Design

This was a single-arm, open-label, single-center switching study conducted at Chi Mei Hospital, Liouying, Tainan City, Taiwan. The study was performed in accordance with the current version of the Declaration of Helsinki (52^nd^ WMA General Assembly, Edinburgh, Scotland, October 2000) and in agreement with the International Conference on Harmonisation (ICH) guidelines on Good Clinical Practice (GCP). All patients provided written informed consent to participate in the study prior to being screened. The study was performed in compliance with the requirements of the Chi Mei Medical Center Institutional Review Board. The protocol and ICF were approved by the Institutional Review Board on 25 September 2017, and revisions were approved on 14 June 2018.

### 2.2. Inclusion Criteria

The study included adult patients aged 20 years or older with POAG whose IOP did not exceed 22 mmHg at baseline. The patients were treated with topical prostaglandin ophthalmic solution (0.005% latanoprost) for more than 3 months without dry eye therapy. All of them had corneal disorders before enrollment in at least one eye (at least one eye had a score above 1 on the National Eye Institute/Industry [NEI] scale) and were followed up on an outpatient basis. When both eyes were eligible, the eye with a higher NEI score was selected for evaluation.

### 2.3. Exclusion Criteria

Patients with any of the following conditions were excluded: severe visual field disorder (mean deviation of 15 dB or worse), received corneal refractive surgery, history of ocular surgeries within 3 months prior to enrollment, any corneal abnormality or other condition preventing IOP measurement, use of artificial tears to relieve dry eye symptoms, ocular allergy, ocular infection or ocular inflammation which may affect the interpretation of the results of the study, use of systemic or ophthalmic steroids (excluding topical skin steroidal ointment) and antiglaucoma agents other than prostaglandin ophthalmic solution, pregnancy or lactation, history of hypersensitivity to the drugs to be used during the study period (anesthetic ophthalmic solution, fluorescein, etc.) or to the drugs similar to the study medication (the PGAs other than tafluprost), use of contact lenses during the study period, or participation in another clinical trial involving an investigational drug/device or participation in such a trial within the last 30 days.

### 2.4. Procedures

All included patients had been on treatment with topical ophthalmic latanoprost 0.005% for at least 3 months before enrollment. All patients have switched to tafluprost 0.0015% ophthalmic solution on visit 0 (Day 0), at a dose of one drop per affected eye once daily. Patients were instructed to instill the study drug in the evening during the treatment period until visit 2 (week 12 ± 2 weeks). The concomitant use of ophthalmic medications and treatments, except over-the-counter drugs not containing BAK, was prohibited throughout the study period.

### 2.5. Outcome Measurements

The primary outcome was the change in fluorescein staining score by NEI at visit 2 from baseline. The secondary outcomes were the change in the IOP at each visit, change in the fluorescein staining score at visit 1 (week 4 ± 2 weeks), changes in ocular symptoms at each visit, changes in tear break-up time (TBUT) at each visit, changes in hyperemia severity at each visit, and other adverse drug reactions.

The corneal assessment (fluorescein staining), IOP measurement, TBUT, assessment of hyperemia, ocular symptoms, and other adverse drug reactions were all measured at visits 0, 1, and 2. The corneal evaluation was performed by using the NEI method [15]. In this method, the cornea was divided into five areas (central, superior, inferior, temporal, and nasal). The cornea was stained with fluorescein test paper, and the staining degree seen under slit-lamp microscopy in each area was given a corresponding score as follows: 0 = none; 1 = sparse; 2 = dense, and 3 = coalesced. The NEI score was the sum of scores from the five areas.

IOP was measured using the method applied in clinical practice by using a Canon TX-20P Full Auto Tonometer (Anon Components, Inc.; Saitama, Japan). During TBUT assessment, the tear film was stained by using fluorescein dye, and the cornea was observed under cobalt blue light; the time (in seconds) until the tear film broke and the corneal surface was exposed under slit-lamp microscopy was then measured. Ocular symptoms of irritation/burning/stinging, foreign body sensation, tearing, itching, and dry eye sensation were assessed using a patient questionnaire, which graded symptom severity as follows: 0 for none, 1 for trace, 2 for mild, 3 for moderate, and 4 for severe. Hyperemia was assessed by examination of the bulbar and palpebral conjunctiva and categorized by severity as follows: None (no manifestations), mild (dilation of some vessels), moderate (dilation of most vessels), and severe (vasodilatation of all vessels of the bulbar conjunctiva or individual blood vessels of the palpebral conjunctiva nondistinguishable). The proportion of patients in each category was calculated.

On each visit, patients answered a questionnaire to assess treatment compliance. The questionnaire utilized a 4-point scale as follows: everyday (100%), sometimes forgot to use (75%–99%), used in approximately half the number of days (25–74%), and rarely used (<25%). The proportion of patients indicating each category was reported.

All measures were summarized descriptively. Continuous variables were summarized as means and standard deviation. Categorical variables were tabulated by frequency (*n*) and percent (%). There was no statistical analysis plan for this study due to the small sample size. However, post-hoc statistical testing was conducted on some endpoints by using paired *t*-test to provide an additional understanding of data trends.

## 3. Results

### 3.1. Patients

Twenty patients provided informed consent and were enrolled in the study, while 3 patients discontinued early due to mild adverse events (For example, eye itchiness, headache, and fatigue, respectively). Seventeen patients completed the study; the mean age was 62.2 ± 10.6 years, and 11 patients (64.7%) were male (Table 1). The baseline IOP of the study eyes was 13.1 ± 14.5 mmHg. The baseline NEI score was 7.1 ± 3.2. Table 1 summarizes the baseline characteristics of the included patients. The other reported BAK-containing concomitant ophthalmic medications were sulfamethoxazole (SINOMIN; Shionogi Co Ltd, Osaka, Japan) (58.8%), pirenoxine (KARY UNI; Santen Pharmaceutical Co. Ltd, Osaka, Japan) (47%), and cyanocobalamin (SANCOBA; Santen Pharmaceutical Co. Ltd, Osaka, Japan) (23.5%) (Table 1). The most commonly reported oral medication was tamsulosin (11.7%).

### 3.2. NEI Score

The mean NEI score of the entire cornea was 7.1 ± 3.2 at baseline and 5.3 ± 1.8 at visit 2 (Table 2), for a mean difference of −1.8 ± 2.2 (*p* < 0.01). Compared with baseline, 11/17 patients (64.7%) had improved NEI scores, and 6/17 (35.3%) had no significant change. The mean NEI change from baseline to visit 1 was also statistically significant (−1.5 ± 2.3; *p* < 0.05). At visit 1, 9/17 patients (52.9%) had improved NEI scores, 7/17 (41.2%) had unchanged NEI scores, and one patient had a slight increase.

### 3.3. IOP

**Figure 1:**
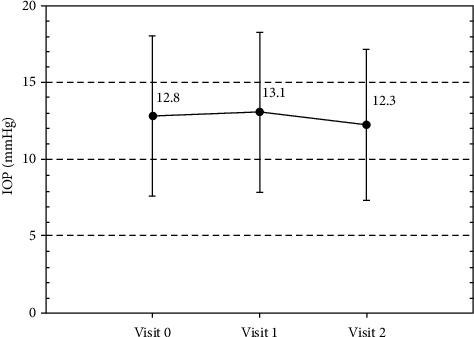
Mean intraocular pressure at each visit.

The mean IOP at baseline was 12.8 ± 4.6 mmHg, and IOP control was maintained, with no significant changes observed throughout the study period (Figure 1). The mean IOP at the end of the study was 12.3 ± 4.0 mmHg (*p*=0.470).

### 3.4. Hyperemia, TBUT and Ocular Symptoms

**Figure 2:**
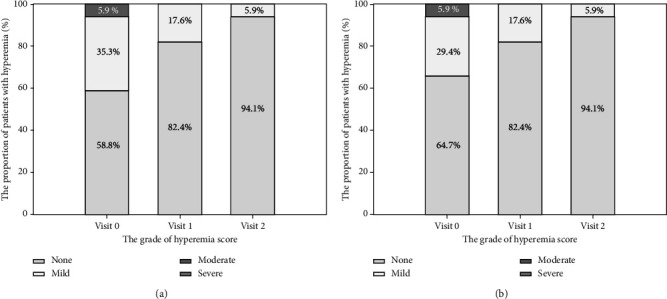
proportion of patients by hyperemia severity per visit. (a) Bulbar conjunctiva. (b) Palpebral conjunctiva.

The proportion of patients with no signs of hyperemia on the bulbar conjunctiva increased from 58.8% (baseline) to 82.4% on visit 1, and 94.1% on visit 2 (Figure 2). Similarly, the proportion of patients with no signs of hyperemia on the palpebral conjunctiva increased from 64.7% (baseline) to 82.4% on visit 1, and 94.1% on visit 2. Compared with baseline, TBUT increased by 40.8% (0.8 ± 1.5 s; p < 0.05) on Visit 1, and 70.6% (1.2 ± 1.7 s; p < 0.05) on Visit 2. The mean score in dry eye sensation significantly decreased from baseline to visit 2 by 0.5 ± 0.8 (*p* < 0.05), with 58.8% (10/17) reported improvement, 29.4% (5/17) with the unchanged grade, and 11.8% (2/17) with worsening. The respective changes in scores for irritation/burning/stinging, itching, foreign body sensation, and tearing from baseline to visit 2, were −0.4 ± 1.4, -0.1 ± 0.6, −0.1 ± 0.9, and −0.1 ± 0.5, respectively (Table 3), which were all not statistically significant.

### 3.5. Adverse Events

Aside from the three mild adverse events previously mentioned, which led to early treatment discontinuation, no other adverse events were reported. No deaths or serious adverse events were reported. Assessment of treatment compliance showed that at baseline, 19/20 of patients (90.0%) reported full compliance to medications. At visits 1 and 2, 16/18 (88.9%) and 15/17 (88.2%), respectively, reported full daily compliance.

## 4. Discussion

The treatment of glaucoma often requires prolonged application of topical IOP-lowering medications. However, this is also associated with clinical signs of OSD due to prolonged exposure to BAK, a quaternary ammonium cationic surface-acting agent that acts as a detergent on cell membranes and is commonly used as a preservative [7–9, 16]. Latanoprost eye drops commercially available in Taiwan contain 0.02% BAK [7–9]. The corneal disorders associated with BAK have been attributed to modification and/or disruption of the lipid layer of the tear film, leading to hyperemia, punctate keratitis, dry eye, or irritative ocular symptoms [7–9].

Tafluprost 0.0015% is a PG that has been demonstrated to have similar IOP reduction over a 24-hour period compared with commercially available latanoprost [17]. Furthermore, commercially available tafluprost ophthalmic solution contains 0.001% BAK, which is 20 times lower than commercially available latanoprost eye drops. By reducing the concentration of BAK, it is believed that the adverse effects of this preservative on the ocular surface could be minimized.

The present study showed that POAG patients with corneal disorders (associated with 0.005% latanoprost use) experienced improvements in fluorescein staining scores after switching to 0.0015% tafluprost with reduced BAK. Furthermore, the switch was not associated with loss of IOP control, which is consistent with previous studies with similar study designs [17–19]. This study also found that switching to tafluprost was associated with a 70.6% (1.2 ± 1.7 s; *p* < 0.05) increase in TBUT as well as a lower rate of hyperemia and decreased hyperemia severity on visit 2.

A similar study conducted in Singapore included patients with POAG or OH that had been on 0.005% latanoprost treatment for 3 months and *a* ≥1 NEI ocular surface staining score. The study analyzed data from 51 patients and found that tafluprost treatment for 3 months was associated with a decrease in NEI score of 3.6 ± 2.2 (*p* < 0.001) as well as significant improvements in TBUT (0.7 ± 2.1 s; *p*=0.017), hyperemia score, and subjective symptoms (all *p* < 0.05) [18]. Additionally, patients did not experience a significant change in IOP control (mean change from baseline: −0.1 ± 3.1; *p*=0.874). Another switching study evaluated the effect of switching 28 glaucoma patients from latanoprost or travoprost ophthalmic solution with corneal epithelial disorders to BAK-reduced tafluprost. After 2 months, patients previously on latanoprost or travoprost had significantly improved fluorescein staining scores (*p* < 0.0001and 0.001, respectively) while IOP-lowering efficacy was maintained [13]. A prospective, multicenter, open-label study in Japan has demonstrated the tolerability and efficacy of BAK-reduced tafluprost in treating Japanese glaucoma patients. The study included 30 patients previously treated with preserved PGAs and had SPK. Patients were switched to tafluprost, which was administered for 12 weeks. The patients had a decrease in mean IOP from 15.6 ± 2.6 mmHg to 14.4 ± 2.0 mmHg (*p* < 0.01) [19]. Furthermore, the mean SPK area density score decreased significantly (*p* < 0.0001) while the mean TBUT increased significantly (*p* < 0.01); the mean hyperemia score remained unchanged. Besides, a prospective, randomized crossover study in Egypt was compared.

The efficacy and the tolerability of preservative-free tafluprost and latanoprost. Corneal erosions and conjunctival hyperemia were significantly higher in the latanoprost-treated patients. TBUT worsened significantly after treatment with latanoprost at 2 and 5 months (*p* < 0.001 and *p* = 0.026, respectively) [20].

Real-world evidence also supports the favorable safety and tolerability profile of tafluprost ophthalmic solution. A retrospective study of patients treated with tafluprost 0.0015% in a routine clinical setting in the Philippines, which included 329 eyes in 177 patients, found that the majority of patients (≥85%) reported no adverse effects over a mean follow-up period of 8.8 months. The rates of eye redness and conjunctival hyperemia were 10% and 15%, respectively [21]. No patients discontinued treatment due to adverse symptoms or signs during the study.

The low risk of adverse effects of 0.0015% tafluprost with reduced BAK to the ocular surface may potentially contribute to treatment adherence, which is crucial for chronic conditions such as glaucoma. By the end of the study, 88.2% of patients reported full compliance with treatment. Among the three patients that discontinued, only one complained of ocular symptoms (itchiness), and the severity of adverse events in all three patients was mild. The clinical benefit of reducing BAK exposure may be related to reduced ocular inflammation (For example, lower matrix metalloproteinase [MMP] levels on ocular surfaces, particularly MMP9) and lack of adverse effects on conjunctival goblet cells. A clinical study found that more glaucoma patients on BAK-preserved PGAs had elevated MMP9 in tears (>40 ng/mL) compared with those on BAK-free tafluprost (46.7% vs. 16.7%;*p* < 0.05) [22]. Another study found that patients treated with either BAK-free tafluprost or physiologically buffered saline had no decrease in conjunctival goblet cell density, whereas those on preservative-containing latanoprost had a significant decrease in goblet cell density vs. baseline (*p* < 0.05) [23].

To our knowledge, this is the first study to evaluate tafluprost on a Taiwanese population and the first study in Taiwan to evaluate the effect of switching glaucoma patients from a PG with another drug of the same class. Additionally, we tried to limit the use of concomitant ocular medications to no more than three medications and no other BAK or topical anti-inflammatory drugs to avoid potential interference with the effects of the test drug, including its reduced BAK content, on the condition of the ocular surface. In addition to demonstrating the efficacy and safety of tafluprost in this population, we were also able to describe important characteristics of these patients, including treatment adherence, use of concomitant medications, and reasons for treatment discontinuation. These types of information would be useful in planning future studies, such as sample size calculations and recruitment planning. Nonetheless, the small sample size of the study is an important limitation. The study also initially intended to enroll patients with OH, but no such patient was found eligible after screening. The small sample size and inability to enroll OH patients were related to the stringent inclusion/exclusion criteria. However, this limitation resulted in findings that are focused on patients with POAG, which are the primary target patients of prostaglandins; hence, the applicability of the study is improved. Undoubtedly, challenges in enrollment should be addressed in future studies through multiple study sites and the allowance for a longer recruitment period. Larger, two-arm, double-blind, crossover studies may also be conducted to better evaluate the impact of treatment switching during IOP-lowering therapy.

The study is also limited by the short follow-up period. Longer-term studies up to 2 years are recommended to better detect adverse safety signals and evaluate long-term IOP control and treatment adherence. A real-world, noninterventional, open-label, long-term study that included 4,502 tafluprost-treated patients with glaucoma or OH found that over a 2-year period, mean IOP was significantly reduced from 18.6 ± 5.9 mmHg at baseline to 15 mmHg or below, with adverse reactions (eyelid pigmentation, ocular hyperemia, eyelash changes, eyelid hypertrichosis, and iris hyperpigmentation) observed in 18.64% of patients [24].

## 5. Conclusion

The results of this study showed that switching from BAK-containing medications to BAK-reduced tafluprost 0.0015% significantly improved fluorescein staining score, TBUT, and conjunctival hyperemia while maintaining IOP control.

## Figures and Tables

**Table 1:** Demographics and baseline characteristics of enrolled patients.

Variable	Results (N = 17)
Sex (*n*, %)	
Male	11 (64.7%)
Female	6 (35.3%)

Age (mean ± SD; years)	62.2 ± 10.6

Diagnosis, right eye (n, %)	
POAG	17 (100.0%)
OH	0
Others	0
Duration of diagnosis (mean ± SD; months)	48.8 ± 37.6

Diagnosis, left eye (*n*, %)	
POAG	16 (94.0%)
OH	0
Others	1 (6.0%)
Duration of diagnosis (mean ± SD; months)	45.9 ± 36.9

Study eye (*n*, %)	
Right	9 (53.0%)
Left	8 (47.0%)

Concomitant ocular medications (*n*, %)	
Sulfamethoxazole	10 (58.8%)
Pirenoxine	8 (47%)
Cyanocobalamin	4 (23.5%)
Neostigmine	3 (17.6%)

OH, ocular hypertension; POAG, primary open-angle glaucoma; SD, standard deviation.

**Table 2:** Results of fluorescein staining scores (NEI scale) at each visit.

Location	Visit 0	Visit 1	Visit 2
Central	1.2 ± 0.6	1.1 ± 0.5	1.1 ± 0.4
Change from V0	N/A	−0.1 ± 0.2	−0.1 ± 0.3
*P*-value		0.332	0.164

Superior	1.3 ± 0.8	1.1 ± 0.2	1.0 ± 0.4
Change from V0	N/A	−0.2 ± 0.7	−0.3 ± 0.6
*P*-value		0.164	0.056

Inferior	1.5 ± 0.7	1.1 ± 0.2	1.1 ± 0.2
Change from V0	N/A	−0.4 ± 0.6	−0.4 ± 0.6
*P*-value		<0.05	<0.05

Temporal	1.5 ± 0.7	1.1 ± 0.3	1.1 ± 0.4
Change from V0	N/A	−0.4 ± 0.6	−0.5 ± 0.6
*P*-value		<0.05	<0.01

Nasal	1.6 ± 0.8	1.2 ± 0.4	1.1 ± 0.5
Change from V0	N/A	−0.4 ± 0.6	−0.5 ± 0.6
*P*-value		<0.05	<0.01

Overall	7.1 ± 3.2	5.5 ± 1.4	5.3 ± 1.8
Change from V0	N/A	−1.5 ± 2.3	−1.8 ± 2.2
*P*-value		<0.05	<0.01

NEI, National Eye Institute.

**Table 3:** Results of secondary endpoints at each visit.

Endpoint (mean ± SD)	Visit 0	Visit 1	Visit 2
TBUT (s)	2.9 ± 1.3	3.6 ± 1.3	4.1 ± 1.3
Change from V0	N/A	0.8 ± 1.5	1.2 ± 1.7
*P*-value		<0.05	<0.05

Irritation/burning/stinging score	0.7 ± 1.1	0.2 ± 0.4	0.3 ± 0.6
Change from V0		−0.5 ± 1.1	−0.4 ± 1.4
*P*-value		N/A	N/A

Itching score	0.2 ± 0.6	0.2 ± 0.6	0.2 ± 0.4
Change from V0		−0.0 ± 0.7	−0.1 ± 0.6
*P*-value		N/A	N/A

Foreign body sensation score	0.3 ± 0.8	0.2 ± 0.6	0.2 ± 0.4
Change from V0		−0.1 ± 1.1	−0.1 ± 0.9
*P*-value		N/A	N/A

Tearing score	0.3 ± 0.6	0.1 ± 0.5	0.2 ± 0.4
Change from V0		−0.2 ± 0.8	−0.1 ± 0.5
*P*-value		N/A	N/A

Dry eye sensation score	0.8 ± 0.8	0.4 ± 0.7	0.3 ± 0.5
Change from V0		−0.5 ± 0.9	−0.5 ± 0.8
*P*-value		<0.05	<0.05

## Data Availability

The clinical data used to support the findings of this study are available from the corresponding author upon request.
